# WebTetrado: a webserver to explore quadruplexes in nucleic acid 3D structures

**DOI:** 10.1093/nar/gkad346

**Published:** 2023-05-09

**Authors:** Bartosz Adamczyk, Michal Zurkowski, Marta Szachniuk, Tomasz Zok

**Affiliations:** Institute of Computing Science and European Centre for Bioinformatics and Genomics, Poznan University of Technology, Piotrowo 2, 60-965 Poznan, Poland; Institute of Computing Science and European Centre for Bioinformatics and Genomics, Poznan University of Technology, Piotrowo 2, 60-965 Poznan, Poland; Institute of Computing Science and European Centre for Bioinformatics and Genomics, Poznan University of Technology, Piotrowo 2, 60-965 Poznan, Poland; Institute of Bioorganic Chemistry, Polish Academy of Sciences, Noskowskiego 12/14, 61-704 Poznan, Poland; Institute of Computing Science and European Centre for Bioinformatics and Genomics, Poznan University of Technology, Piotrowo 2, 60-965 Poznan, Poland

## Abstract

Quadruplexes are four-stranded DNA/RNA motifs of high functional significance that fold into complex shapes. They are widely recognized as important regulators of genomic processes and are among the most frequently investigated potential drug targets. Despite interest in quadruplexes, few studies focus on automatic tools that help to understand the many unique features of their 3D folds. In this paper, we introduce WebTetrado, a web server for analyzing 3D structures of quadruplex structures. It has a user-friendly interface and offers many advanced features, including automatic identification, annotation, classification, and visualization of the motif. The program applies to the experimental or *in silico* generated 3D models provided in the PDB and PDBx/mmCIF files. It supports canonical G-quadruplexes as well as non-G-based quartets. It can process unimolecular, bimolecular, and tetramolecular quadruplexes. WebTetrado is implemented as a publicly available web server with an intuitive interface and can be freely accessed at https://webtetrado.cs.put.poznan.pl/.

## Graphical Abstract

**Graphical Abstract:**
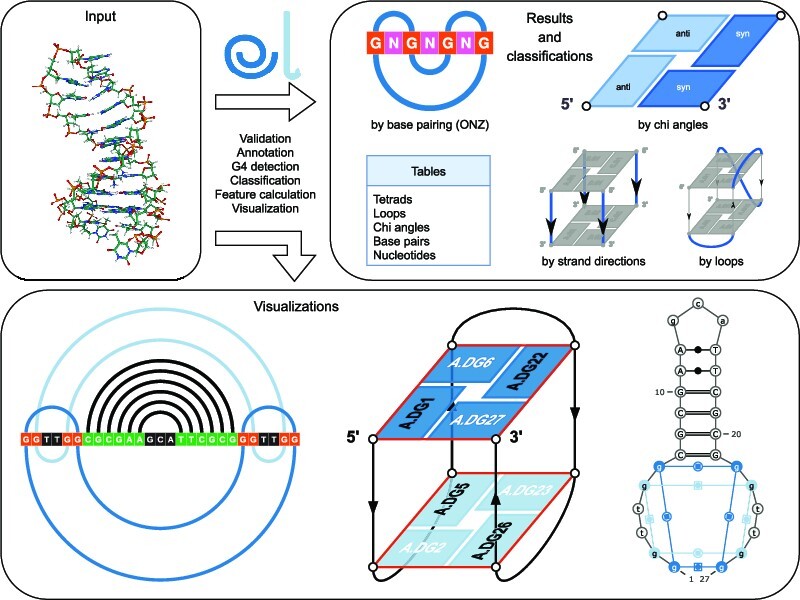
WebTetrado: a webserver to explore quadruplexes in nucleic acid 3D structures.

## INTRODUCTION

Quadruplexes are four-stranded DNA and RNA motifs that form in genomic regions rich in guanine. They are involved in many genomic processes, including transcription, replication, and epigenetic regulation (1). Numerous studies point to their association with the growth and progression of cancer and other diseases. All this makes quadruplexes promising targets in drug design and interesting subjects of structural studies (2–5).

In 2020 Popenda *et al.* proposed a classification scheme derived from base pairing patterns in tetrads (6). They defined three classes, O, N and Z, named after the shape observed in the tetrad visualizations. Each class has clockwise and anticlockwise progression, indicated with + and −, respectively. Next, they proposed classifying a quadruplex as O, N or Z if all of its tetrads are of this type or M (mixed) otherwise. In addition, they added a suffix p, a or h for the parallel, antiparallel, or hybrid orientation of the strands, respectively.

The topologies underlying the classification of quadruplexes and other parameters of their structures can be analyzed using a few computational tools. DSSR (7) was the first to target the detection of G-quadruplexes in 3D structure data saved in PDB and PDBx/mmCIF files and to describe their features. It runs systematically on all entries in the Protein Data Bank and collects motifs found in the DSSR-G4DB database. ElTetrado (8) can identify and analyze G4s and other kinds of tetrads and quadruplexes, classify them, and compute their parameters. It is the core of the computation pipeline running within the ONQUADRO database system (9). The most recent tool for processing atom coordinates in the search for quadruplexes is ASC-G4 (10). It calculates more features than DSSR and ElTetrado, but is limited to unimolecular quadruplexes and supports only the PDB format.

In this paper, we introduce WebTetrado, a web server for analyzing 3D structures of quadruplexes. It has a user-friendly interface and offers many new features compared to its command-line predecessor, ElTetrado. Novelties include dedicated visualizations thanks to tight integration with our advanced tool DrawTetrado (11).

## METHOD OUTLINE

**Figure 1.**
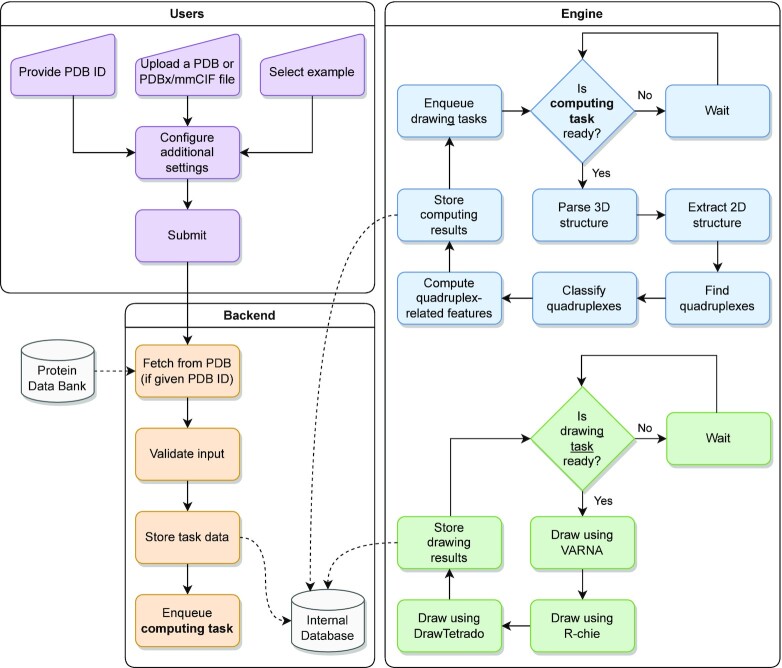
WebTetrado workflow.

The first step in the WebTetrado pipeline (see Figure 1) is to read the input data and the configuration parameters. The front-end feeds these data to the back-end on the basis of the input form on the main page. The validation protocol ensures that the main input is a correct PDB or PDBx/mmCIF file and that all other analysis parameters have viable values. The back-end stores successfully validated inputs in a database and enqueues a computing task. This step involves the generation of a unique identifier, which the front-end embeds in a URL. Initially, the URL displays a loading page with the option to turn on browser notification upon the task’s successful completion. Later, the same URL shows the results for the next seven days, after which it expires.

The WebTetrado engine supports parallel processing, so it can handle multiple requests at the same time. The central part of its pipeline starts with reading the configuration metadata from the database. The 3D structure is then loaded and interpreted in terms of its chain, residue, and atom composition. This includes the calculation of the glycosidic bond angle and the classification of each nucleobase as *anti* or *syn*. Next, WebTetrado applies geometrical rules (i.e. constraints on atomic distances, planar and (pseudo)torsion angles) to find stacking and base-base interactions together with their Leontis-Westhof classification. The result of this step allows for the building of a directed graph of nucleotide interactions in which cycles of length four correspond to tetrads in the analyzed structure. This leads to the next step in which the stacking information determined previously is applied on top of the tetrads to locate the N4 helices. Based on chain composition rules, these are divided into distinct quadruplexes, for which WebTetrado traces loop progression and strand connectivity. Moreover, the engine recognizes cations, which play significant roles in quadruplex stability, and proceeds to analyze their proximity to tetrad centers or external sites. Next, the engine classifies the tetrads and quadruplexes according to all its supported schemes and computes quadruplex-related features such as inter-tetrad twist, rise, or planarity deviation. Finally, the quadruplex motif is represented in the two-line dot-bracket format.

These results are stored in the WebTetrado database and a separate drawing task is added to the queue for each supported visualization tool, VARNA (12), R-Chie (13) and DrawTetrado (11). This approach allows for the parallel preparation of all static visualizations. Each drawing task starts by reading the metadata and the computing task’s results from the database.

The VARNA-based procedure uses a set of in-house modifications on top of VARNA software to apply custom coloring and Leontis-Westhof visual annotation, making the quadruplex visualization clear. WebTetrado precomputes four variants of VARNA-based visualization: (i) with interactions constituting tetrads only, (ii) with the addition of canonical pairs outside tetrads, (iii) with all non-canonical interactions and (iv) with all canonical and non-canonical interactions.

The R-Chie-based visualization draws arcs above and below the sequence to display two simultaneous interactions for every in-tetrad nucleotide. This is necessary because G-quartets are based on multiplet base pairing patterns (i.e. each in-tetrad nucleotide has two interacting partners). WebTetrado precomputes two R-Chie-based variants, with and without canonical base pairs outside the tetrads. Unlike tetrad-involved interactions, which use distinct colors for every ONZ class, the arcs representing canonical base pairs are black.

The last tool—DrawTetrado—is coupled the most with WebTetrado, as the computing task’s results directly influence its working. DrawTetrado prepares a 2.5D view of each G4-helix and quadruplex, showing stacking information, *anti*/*syn* conformation, and loop progression.

## WEB APPLICATION

WebTetrado consists of three modules designed to provide flexibility and stability. The service core (engine) is responsible for processing user requests. It is built on top of the lightweight Flask server framework and integrates the ElTetrado tool (8) to identify and process quadruplex data. The next module, the back-end, uses database-driven middleware to manage, queue, and store user requests. It uses the Django web server framework (version 4.1) and the Redis task queue broker, enabling fast processing of concurrent workloads. The engine and the back-end use a Python 3.10 environment with dedicated bioinformatics libraries. They communicate via an OpenAPI-specified interface, which allows automatic validation. The web-accessible front-end is based on TypeScript’s React 17 framework, extended with ant-design components. It provides a series of structure visualizations prepared with four incorporated graphical tools: VARNA (12), R-Chie (13), DrawTetrado (11) and Mol* (14). We designed WebTetrado to work on any modern web browser, either mobile or desktop. It is hosted and maintained by the Institute of Computing Science, Poznan University of Technology, using the Docker container service.

### Input and output description

The input for WebTetrado is the tertiary structure of the nucleic acid given as the atomic coordinates in a PDB or PDBx/mmCIF file. Users upload the file from a local drive or provide the PDB id of a structure. In the latter case, the back-end automatically downloads the corresponding file from the Protein Data Bank (15). Six ready-to-use examples are also available in the system to familiarize users with the tool’s capabilities. Additional settings condition the identification of tetrads and quadruplexes in the input structure and their classification. We provide sensible defaults, but optionally users can modify their values.

Users can select a particular model to analyze in the case of a multi-model input file. Next, they can instruct the system to turn off G-tetrads highlightning, i.e. canonical ones composed of exactly four guanines. By default, WebTetrado does not make assumptions about nucleotide composition and finds all types of quartet, but it highlights the canonical G4s among them. This behavior can be disabled. In addition, the next setting controls whether tetrads are detected with cWH pairings only. Again, these pairings are present in the usual G4 tetrads, but by default WebTetrado generalizes the search for quartets and looks for all kinds of pairs between in-tetrad nucleobases. In addition, users can set how many nucleotides to accept for stacking mismatch. It controls how sensitive WebTetrado should be to inherent uncertainty in stacking interaction detection. In a perfect, canonical quadruplex, each tetrad pair contains four pairs of stacked nucleobases. However, for several reasons, this might not be detected as such. For example, if the structure resolution is low or if it is an intermediate stage taken from the molecular dynamics trajectory, then most likely not all four nucleobase pairs will be recognized as stacked. To alleviate this issue, WebTetrado makes it possible to set a mismatch threshold. By default, at least two pairs of nucleobases stacked between tetrads allow them to be treated as part of the same quadruplex. Finally, users can disable chain reordering, required to classify bi/tetramolecular quadruplexes, which is enabled in default runs. Keeping the original order of chains, as given in the PDB or PDBx/mmCIF file, depends on the input settings.

The result page has a dedicated, bookmarkable URL that allows users to return up to 7 days after completing the task. It displays all gathered quadruplex-related information and visualizations: (i) metadata concerning the structure (PDB id, molecule type, experimental method), (ii) the sequence of the input molecule and its secondary structure in a two-line dot-bracket with colored G-tracts, (iii) quadruplex description (sequence, number of tetrads, type by number of strands, loop description, tetrad combination, rise, twist, type by strand orientation, ONZM class), (iv) tetrad description (sequence, nucleotides, planarity, χ angles, base pairs with Leontis-Westhof classification, ONZ class) and (v) visualizations of the secondary and tertiary structures with ONZ-related coloring (classical, arc and layer diagrams, a cartoon model).

Users can download the results in CSV format for tabular data and SVG or PNG formats for 2D and 3D structure visualizations.

## RESULTS AND DISCUSSION

### User interface

**Figure 2.**
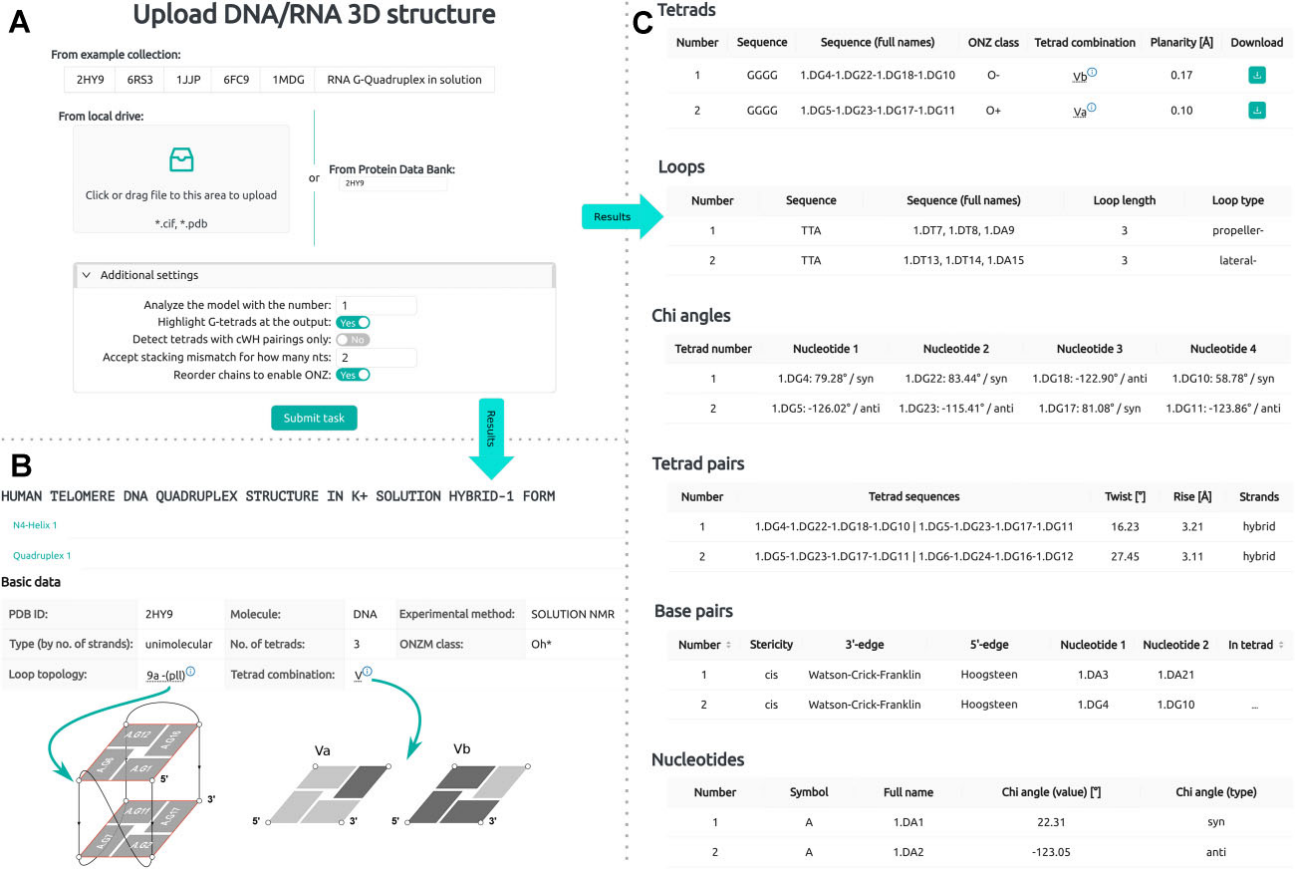
User interface of WebTetrado: (**A**) submission form, (**B**) result summary, (**C**) tables with detailed data.

Figure 2 shows screenshots of the WebTetrado service. Panel 2A shows the screen of the submission form, which allows specifying structure calculations. Submitting a task redirects to a self-refreshing waiting page, allowing users to enable browser notifications. If enabled, the browser will show a message when WebTetrado finishes processing the request.

The remaining panels are the main parts of the result page. The panel 2B shows a table with general information about a quadruplex. Above the table, two tab selectors make it possible to show a different N4 helix or a different quadruplex. Panel 2C shows the content of the result page. It includes several tables with details about tetrads, loops, χ angles, tetrad pairs, base pairs and nucleotides.

### Analysis of the major G-quadruplex form of HIV-1 LTR

**Figure 3.**
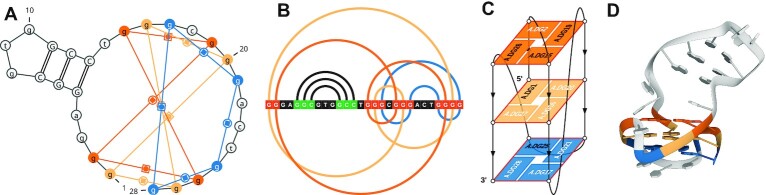
Major G-quadruplex form of HIV-1 LTR (PDB id: 6H1K) visualized in WebTetrado: (**A**) 2D diagram with a visible stem-loop, (**B**) arc diagram with stem–loop as black arcs, (**C**) 2.5D visualization allowing to trace the quadruplex fold, (**D**) 3D image color-coded as the other ones.

G-quadruplex-forming sequences are widespread in genomes, including viral ones. Human immunodeficiency virus 1 (HIV-1) has a 5’-LTR (long terminal repeat) promoter, which plays an important role in the viral replication cycle and is regulated by G-quadruplexes (16). In particular, the LTR-III fragment forms the most stable G-quadruplex. In 2018, Butovskaya *et al.* reported the NMR structure of LTR-III in a K+ solution and deposited it in the Protein Data Bank with PDB id 6H1K (17). The reported structure has several unique and difficult-to-identify features, all of which the WebTetrado can find. First, it contains an elongated loop that folds into a stem-loop motif, making the entire structure a quadruplex-duplex combination (see Figures 3A, B and D). Such quadruplex-duplex motifs have been actively investigated due to their features and potential applications in medicine and biotechnology (18). Furthermore, the HIV-1 LTR-III quadruplex includes a V-shaped loop, which occurs when the 5’-endmost tetrad lies in the middle of the G-quartet stack (see Figure 3C). In addition, it has a hybrid pattern of strand orientations and a combination of 1 nt propeller, 3 nt lateral and 12 nt diagonal loops (see Figure 3C).

The VARNA and R-Chie visualizations are semi-interactive—the user may reconfigure them using switches placed above them in the user interface. These switches change the visibility of base pairs outside the tetrads. In particular, for the HIV-1 LTR-III quadruplex, the visualization of the duplex fragment can be disabled to focus only on the quadruplex part. All four visualizations are color-coded according to the ONZ scheme, which makes it easier to understand the tetrad features in different contexts.

WebTetrado automatically finds all the confirmations of the unique quadruplex topology in the 6H1K PDB structure. In addition, it classifies the tetrads and quadruplex according to Webba da Silva (19) and the ONZ scheme (6). According to it, the HIV-1 LTR-III structure contains two Z and one O tetrad, making it an Mh (mixed hybrid) class quadruplex. The mixed class encompasses the rarest and most complex quadruplex topologies. WebTetrado also computes several quantitative features of the G4 and shows the structural data: nucleobase conformations and base-pairing information both in the tetrads and in the stem–loop motif.

## CONCLUSIONS

WebTetrado is a new web server for analyzing structures containing quadruplexes, four-stranded DNA/RNA motifs of high functional significance that fold into complex shapes. It supports automatic identification and advanced analyses of all types of quadruplexes based only on atomic coordinates. WebTetrado provides a wealth of data computed from the given input file, including classification schemes recognized by the G4 community. In addition, it shows visualizations specially designed to represent quadruplexes. The tool is free and open to anyone interested in the analysis of DNA/RNA structures that include quadruplex motifs.

## DATA AVAILABILITY

WebTetrado is implemented as a publicly available web server with an intuitive interface and can be freely accessed at https://webtetrado.cs.put.poznan.pl/.
